# Human Sperm Bioassay for Reprotoxicity Testing in Embryo Culture Media: Some Practical Considerations in Reducing the Assay Time

**DOI:** 10.1155/2010/136898

**Published:** 2010-12-19

**Authors:** Amjad Hossain, Subhash Aryal, Collin Osuampke, John Phelps

**Affiliations:** ^1^Division of Reproductive Endocrinology, Department of Obstetrics and Gynecology, The University of Texas Medical Branch, 301 University Boulevard, Galveston, TX 77555, USA; ^2^Department of Biostatistics, Health Science Center, University of North Texas, Fort Worth, TX 76107, USA

## Abstract

Human sperm assay (HSA) is a preferred in house quality control and proficiency test (PT) practiced in fertility laboratories. HSA is performed over varying durations, apparently without following set criteria. To better understand the assay time required for reprotoxicity testing in embryo culture media, we compared American-Association-of-Bioanalysts-(AAB-) administered HSA data to our own assay performed using PT samples obtained from AAB. Participating laboratories were required to culture sperm for 48 hours to determine media acceptability. Conclusions drawn from 48- and 24-hour observations were the same, suggesting that HSA could identify reprotoxic media in less time than required by AAB. Our assay revealed that changes in motility grade in adulterated media are significantly different from those in control media. Furthermore, grade changes can be identified earlier than differences in motility loss between samples. Analyzing motility and motility quality together provides a method for establishing an optimal time for HSA.

## 1. Introduction

Quality control (QC) is an essential component of a successful human in vitro fertilization (IVF) program. The success of IVF depends critically on the quality of the products used in the laboratory procedures. The reprotoxicity testing of reagents, media, and consumables utilized in the fertility laboratory is therefore essential [1–4]. Accordingly, numerous bioassays have emerged over time for evaluating procedural quality [5–8]. 

Among these bioassays, the human sperm assay (HSA) has been an integral part of IVF since its inception. Edwards and Steptoe, the 2 pioneers of human IVF, utilized HSA for testing the suitability of the solutions and materials used in the first successful IVF pregnancy in the world [1, 9]. The sperm assay allowed them to detect factors that adversely affected the growth of human embryos in vitro, thus offering an opportunity for optimizing the embryo culture conditions [1, 8, 9]. Subsequently, many other assays have been developed utilizing mouse embryos, hamster sperm, ovarian cells, cumulus cells, and so forth, in evaluating the quality of the embryo culture procedures [6–11]. HSA, however, remains one of the preferred QC methods in fertility laboratories where it is routinely used as an in house QC test and also as an externally administered proficiency test (PT).

Review of the HSA literature indicates that human sperm bioassays are being performed over varying durations of time [3, 12–14]. Alvarez proposed 4 hours for the sperm stress test, while in regular sperm bioassays, assay times of 24 to 96 hours have been used [15–20]. The American Association of Bioanalysts (AAB), the largest PT provider for fertility laboratories in USA, chose an assay time of 48 hours for identifying the quality of the embryo culture media [3, 20–22]. It is possible that if an assay is extended beyond the required time, its sensitivity and specificity can be compromised [10, 12, 17, 23–25]. 

In this study, we explored AAB-administered PT to develop a better understanding of the time required for HSA in AAB-set culture conditions. The AAB provides participating laboratories with 2 media samples in each PT event. The quality of one media is intentionally made poorer than that of the other. The laboratories are required to perform HSA for 48 hours to identify the media as being of acceptable (MAQ) or unacceptable (MUQ) quality. The purpose of this PT is for fertility laboratories to develop the skills needed for evaluating the quality of their own media to be used in IVF procedures. The AAB has been administering this PT for more than 10 years. We evaluated the AAB-compiled HSA data for the past 2 years and also performed our own assay using a set of AAB-provided PT samples. We are able to demonstrate that the assay time for HSA utilized in AAB PTs is lengthy, and that evaluation of the motility grade, along with the motility, enhances the efficiency of the assay, therefore helping to optimize the assay time.

## 2. Materials and Methods

### 2.1. AAB-Administered PT Data on HSA

The data of AAB-conducted PT for determining the suitability of culture media utilizing HSA was used (data from AAB: http://www.aab-pts.org/) [20, 21]. The PT score represented human sperm survival in the 2 culture media, labeled embryo culture 1 (EC1) and embryo culture 2 (EC2), in each PT event of year 2008 and 2009. There was a total of 4 events (08 − *E*1, 08 − *E*2, 09 − *E*1, and 09 − *E*2), in each of which EC1 and EC2 were identified either as MAQ or MUQ by performing HSA.

More than 130 fertility laboratories across the United States participated in the AAB-administered PT. The participating laboratories recorded sperm motility as an indicator of sperm survival in EC1 and EC2 at 0, 24, and 48 hours in order to reveal the differences in their quality. The methods used in AAB-administered HSA fall under 2 categories: sperm culture with and without oil overlay. The laboratories were also required to culture sperm with and without protein supplementation.

Statistical analysis of the AAB-compiled sperm motility data (average ± SD) in EC1 and EC2 reported by the participating laboratories (*n* ≥ 130) was performed to see if the motility difference between EC1 and EC2 was significant at 24 and 48 hours. The 95% confidence interval for the difference in means between the 2 media was calculated (EC1 versus EC2) in each PT event (08 − *E*1, 08 − *E*2, 09 − *E*1, and 09 − *E*2). The level of significance (alpha) was adjusted using the Bonferroni correction to account for multiple comparisons, and a *t*-test utilizing pooled variance was used.

### 2.2. HSA Utilizing Motility and Motility Quality

A separate HSA experiment of our own was performed using AAB-provided 09-E2 PT samples. In this experiment, sperm culture in EC1 and EC2 were established following a no oil overlay method of culture in which sperm motility, as well as motility quality, were recorded sequentially at 0, 6, 12, and 24 hours. 

The gradient-washed sperm samples (*n* = 6), exhibiting ≥90% motility and a motility grade predominantly of 4, were used for the convenience of experiment. The cultures, composed of EC1 and EC2 media of 1.0 mL volume containing 4-5 × 10^6^/mL sperm, were established in 5 mL Falcon culture tubes (Becton and Dickinson). All culture conditions were maintained in duplicates in a gas (6% CO_2_) and temperature (37°C) controlled incubator. Sperm motility and motility grade in the culture were evaluated at the indicated time points by assessing aliquots of samples in the Makler chamber following WHO criteria but with a modified grading system [24, 26, 27]. The relative abundance of motile sperm exhibiting different motility grades (G1: nonprogressive; G2: sluggish progressive; G3: progressive; G4: rapid progressive) was documented.

## 3. Results

The AAB data that was brought under our investigation is shown in Table 1. As evident from the motility scores documented in Table 1, the EC1 was unacceptable (MUQ) in PT events 08 − *E*1, 08 − *E*2, and 09 − *E*1, while the EC2 was MUQ in the 09-E2 event.

**Figure 1:**
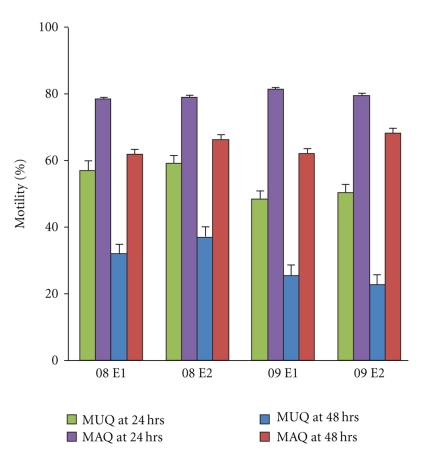
Motility in MUQ and MAQ at 24- and 48-hours observations in the 4 consecutive PT events. The difference between MUQ and MAQ was significant (*P* < .05) in all events at 24 hours as well as 48 hours. MUQ at 24 hours (green); MAQ at 24 hours (purple); MUQ at 48 hours (Blue); MAQ at 48 hoyrs (red).

**Figure 2:**
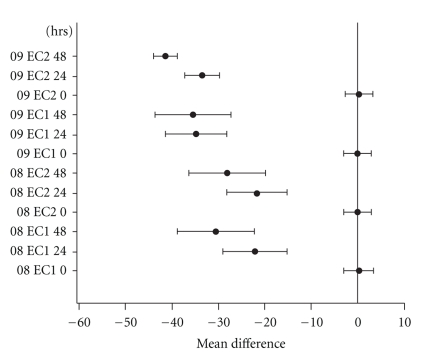
A collection of 95% confidence intervals for the difference in mean motility between 24-hour and 48-hour observations.

The motility difference between EC1 and EC2 (categorized either as MAQ or MUQ) was statistically significant (*P* ≤ .05) at 24 hours as well as at 48-hour observations in all PT events (08 − *E*1, 08 − *E*2, 09 − *E*1, and 09 − *E*2) of years 2008 and 2009 (Figures 1 and 2). As seen in Figure 1, the difference between MUQ and MAQ (EC1 versus EC2) during the first 24 hours was convincingly acceptable in all 4 PT events (08 − *E*1: 51.9 ± 26.0 versus 76.1 ± 14.8; 08 − *E*2: 52.2 ± 24.6 versus 75.2 ± 16.1; 09 − *E*1: 40.7 ± 26.2 versus 77.6 ± 12.4; 09 − *E*2:76.3 ± 11.5 versus 41.8 ± 9.0). Further, the significant difference between EC1 and EC2 at 24 hours was revealed by both assay methods (oil overlay: 38.0 ± 22.8 versus 73.5 ± 14.1; no oil overlay: 49.6 ± 22.0 versus 77.3 ± 14.0) and independently of protein supplementation (with protein: 47.3 ± 22.3 versus 77.5 ± 13.1; without protein: 40.1 ± 22.4 versus 73.1 ± 15.0).

**Figure 3:**
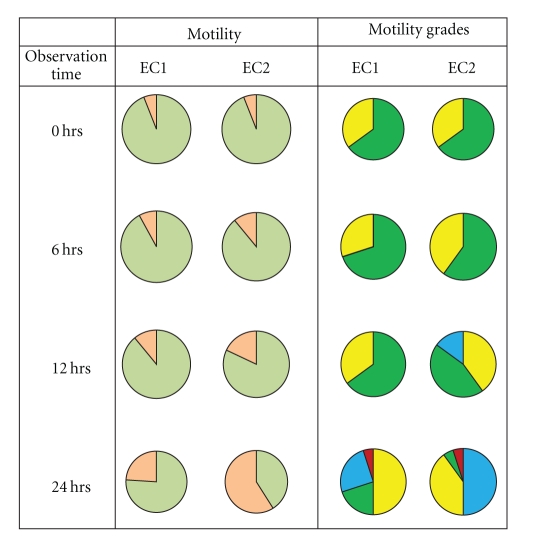
Change pattern in motility (motile: olive green; nonmotile: light orange) and motility grades (G4: green; G3: yellow; G2: blue; G1: red) in AAB-PT event 09 − *E*2 samples (EC1 and EC2) during sperm culture for 24 hours.

The results of our own HSA study are shown in Figure 3. The EC1 and EC2 were identified as MAQ and MUQ, respectively. The presence of toxicant, as in EC2 (MUQ), was better predicted by motility grade than by motility in the early exposure period (6–12 hours). As seen in Figure 3, there was a shift in motility grade composition (EC1: G4 70%, G3 30% versus EC2: G4 60%, G3 40%) but not motility (EC1: 92 ± 4% versus EC2: 89 ± 6%) in the first 6 hours. Evidence of the difference between EC1 and EC2, reflecting changes in motility grade as well as motility, became stronger at 12 hours. However, the differences in motility grade (EC1: G4 65%, G3 35% versus EC2: G4 40%, G3 50%, G2 10%) compared to motility (EC1: 89 ± 3% versus EC2: 82 ± 7%) were predominant. Such difference became more pronounced between the 2 PT specimens (EC1 and EC2) by 24 hours as evident in motility grade (EC1: G4 20%, G3 50%, G2 25%, G1 5% versus EC2: G4 5%, G3 40%, G2 50%, G1 5%) and also in motility (EC1: 76 ± 9% versus EC2: 41 ± 13%).

## 4. Discussion

The HSA has been an integral part of fertility laboratories for many obvious reasons. First, the assay method is user friendly, requiring less technical skill and equipment than other methods. Animal models like mouse embryo assay (MEA) and hamster sperm motility assay (HSMA), the alternatives to HSA, may be commercially available but are expensive and labor intensive. Most importantly, when human sperm is used, no species differences have to be taken into account in interpreting and validating the outcome of the test. Since the first report of successful human IVF, various modifications have been introduced to improve the IVF techniques, and HSA has played a role in such improvements [2, 9, 15]. Therefore, HSA was not only used by the IVF pioneers, but it has also maintained a permanent footing in IVF laboratories to this day. 

The AAB, being an authorized PT provider, has been collecting HSA data from fertility laboratories for more than 10 years. The participating laboratories are required to perform the assay for 48 hours to fulfill AAB requirements. The cost and labor required for an assay are directly related to the assay time. The longer the assay duration, the more expensive the assay becomes. We are able to show that HSA values in EC1 and EC2 are significantly different at 24, as well as at 48, hours in all 4 PT events conducted in the years 2008 and 2009. We argue that when the difference between EC1 and EC2 can be confidently confirmed by 24 hrs, then prolonging the assay up to 48 hours is not necessary. 

We further argue that the loss of motility in any culture, even one completely free of any harmful elements (toxicants), is expected to occur as time progresses. This natural phenomenon of sperm motility loss in culture may overshadow the real toxicant-induced motility loss, producing erroneous results if the assay time is extended beyond that actually required. It thus appears that there is no gain in prolonging the culture for an additional 24 hours in AAB-administered PTs.

The participating laboratories did not have an option for evaluating motility in the culture before 24 hours in AAB-administered PTs. It is possible that a significant motility difference between EC1 and EC2 may have emerged prior to that time (24 hours) but was missed because of lack of investigation. The preliminary results of our own bioassay support this notion. We documented increased motility loss in EC1 compared to EC2 at 6-hour and 12-hour observations but were unable to validate its statistical significance because of our small sample size. It will be worth investigating our observation further with a larger sample size. 

The evaluation of the quality of the sperm motility (motility grade) is now a routine practice in semen analysis [27]. We realized that such parameters will also be informative if they can be incorporated in the bioassay. In HSMA, the motility grade is given an equal emphasis to that of motility [6]. However, motility grade evaluation was not given any consideration in human sperm bioassay [6, 28, 29]. The HSMA neither gained wide acceptance in human fertility laboratories nor influenced HSA to incorporate motility grade evaluation. In our study, we showed that the mode of change in motility grade (motility quality) in adulterated media (EC2) is significantly different from that of the control media (EC1). The onset of the difference in the motility quality between the 2 media (EC1 and EC2) can be identified earlier than the motility loss, as it is logical that any harmful agent will affect the motility quality first before motility is completely lost. Therefore, the inclusion of motility quality evaluation in HSA will increase its sensitivity and thus will help in identifying the difference earlier. Future HSA studies may refine motility quality evaluation so that its incorporation in the assay can be perfected.

We must admit that motility grading in its current state is subjective, and it is difficult to give it a true quantitative face. There may be concern about the concept of sperm motility grade being used as a tool in sperm bioassay due to the subjective nature of distinguishing between the various grades. Identifying motile and nonmotile sperm is a much more straightforward issue than distinguishing between the various grades of sperm motility. However, difficulty in grading motility should not hinder the benefit we can achieve by incorporating motility grade in the assay. We used a grading scheme of our own—which may not be a perfect one—in placing sperm under different motility grades. A lot is now known about the motility characteristics of mammalian sperm; therefore, developing a consensus on grading motility will be easier than before. In our view, overcoming the problem of grading motility involves emphasizing the importance of the issue, understanding the obstacles in quantifying the grades, and then developing consensus on grading. It is our expectation that in the near future, consensus will be developed toward a unified motility grading method by utilizing the technologist's skill in assessing motility grade both in one's own laboratory and between laboratories. The existing difficulty in grading motility should be conquered to obtain the benefit it can provide.

Uniquely, human sperm can remain in culture for a lengthy time [6, 24, 30]. However, this should not be the reason for choosing longer assay times. The assay time should always be the minimum time required to detect the difference between the control and experimental culture. The impact of primary-target determinants may be obscured by the other unwanted variables if the assay is prolonged. The time for a bioassay may not be a fixed one since it will vary depending on the concentration and nature of the toxicant in the sample to be investigated and the assay procedure to be applied. However, it is important to determine the assay time before the assay is performed. Arbitrarily choosing the time may lead to erroneous conclusions. Careful evaluation of the dynamics of motility quality, in addition to simple motility, can make HSA more effective in determining the quality of the test material.

## 5. Conclusion

Our study found that in AAB-administered PTs, the collection of data at 48 hours was not necessary to identify EC1 and EC2 since the conclusion drawn from 48 hours observation was the same as that of 24 hours. In other words, sperm culture for 24 hours and 48 hours revealed the same conclusion about the quality of EC1 and EC2 media in each of the 4 consecutive PT events investigated. Further, it was revealed that the mode of change in motility quality is different in adulterated media compared to the control media, and that change can be identified earlier than the difference in motility loss between the 2 samples. Thus, it appears that motility and motility quality combined can sharpen the sensitivity of the assay and, thus, can help in determining the minimum time required for the assay. Evaluation of motility grade along with motility seems to strengthen the power of the human sperm bioassay. This technique holds promise in our center and now needs to be validated at additional sites, with the hope that it may shorten the time used by the AAB method.

## Figures and Tables

**Table 1:** AAB-compiled PT data on HSA from the reporting of the participating laboratories for the years 2008 and 2009. The value represents motility (mean ± SD) in respective culture conditions at specified observation points.

PT Event	No. of Labs particip	Method type	Obs points	Culture conditions tested			
				EC1	EC2	EC1	EC2
				Protein (+)	Protein (+)	Protein (−)	Protein (−)
08 − *E*1	34	Oil overlay	0 Hr	89.6 ± 7.6	89.0 ± 8.2	89.4 ± 7.6	89.5 ± 7.5
08 − *E*1	96	No oil	0 Hr	88.4 ± 9.6	88.1 ± 9.7	86.5 ± 10.7	86.1 ± 11.3
08 − *E*1	142	Combined	0 Hr	88.6 ± 9.5	88.3 ± 9.7	87.1 ± 10.5	87.0 ± 10.6
08 − *E*2	32	Oil overlay	0 Hr	89.7 ± 8.4	89.8 ± 8.2	89.0 ± 10.4	90.4 ± 8.1
08 − *E*2	95	No oil	0 Hr	88.2 ± 9.0	88.1 ± 8.8	86.6 ± 10.3	86.2 ± 11.3
08 − *E*2	138	Combined	0 Hr	88.5 ± 9.2	88.4 ± 9.1	87.1 ± 10.6	87.2 ± 10.9
09 − *E*1	35	Oil overlay	0 Hr	88.6 ± 10.0	88.6 ± 10.1	89.0 ± 9.0	88.7 ± 9.9
09 − *E*1	97	No oil	0 Hr	88.3 ± 8.5	88.4 ± 8.5	87.4 ± 8.6	87.3 ± 8.9
09 − *E*1	140	Combined	0 Hr	88.4 ± 8.7	88.5 ± 8.7	87.8 ± 8.8	87.3 ± 9.5
09 − *E*2	33	Oil overlay	0 Hr	88.3 ± 12.0	89.5 ± 11.0	88.1 ± 13.0	88.2 ± 12.0
09 − *E*2	101	No oil	0 Hr	89.6 ± 7.0	89.8 ± 7.0	88.0 ± 9.0	88.5 ± 9.0
09 − *E*2	143	Combined	0 Hr	89.2 ± 9.0	88.9 ± 9.0	88.2 ± 9.0	88.4 ± 9.0
08 − *E*1	34	Oil overlay	24 Hrs	50.8 ± 25.9	76.4 ± 11.9	45.7 ± 29.1	76.2 ± 12.6
08 − *E*1	94	No oil	24 Hrs	57.2 ± 25.4	78.6 ± 15.1	49.4 ± 25.2	73.8 ± 15.0
08 − *E*1	140	Combined	24 Hrs	55.7 ± 25.9	78.5 ± 14.2	48.0 ± 26.1	73.6 ± 15.3
08 − *E*2	32	Oil overlay	24 Hrs	50.8 ± 29.6	77.3 ± 15.8	43.3 ± 25.9	69.8 ± 19.7
08 − *E*2	92	No oil	24 Hrs	59.2 ± 21.0	79.2 ± 13.0	51.2 ± 23.9	71.9 ± 19.7
08 − *E*2	135	Combined	24 Hrs	56.1 ± 24.1	78.4 ± 13.7	48.3 ± 25.1	71.9 ± 18.5
09 − *E*1	34	Oil overlay	24 Hrs	34.1 ± 26.1	75.5 ± 10.3	29.1 ± 25.4	71.2 ± 12.2
09 − *E*1	96	No oil	24 Hrs	48.5 ± 25.5	81.6 ± 10.3	39.9 ± 25.6	76.9 ± 12.8
09 − *E*1	138	Combined	24 Hrs	44.5 ± 26.4	79.4 ± 11.5	36.8 ± 25.9	75.1 ± 13.2
09 − *E*2	32	Oil overlay	24 Hrs	71.8 ± 15.0	27.7 ± 10.0	69.5 ± 15.0	21.9 ± 10.0
09 − *E*2	100	No oil	24 Hrs	79.7 ± 13.0	50.4 ± 15.0	75.8 ± 13.0	40.6 ± 14.0
09 − *E*2	141	Combined	24 Hrs	79.0 ± 12.0	45.3 ± 10.0	73.6 ± 11.0	38.2 ± 8.0
08 − *E*1	32	Oil overlay	48 Hrs	20.5 ± 23.1	53.0 ± 24.3	14.6 ± 18.0	53.1 ± 26.9
08 − *E*1	91	No oil	48 Hrs	32.1 ± 24.4	62.0 ± 24.0	25.6 ± 22.6	57.5 ± 23.0
08 − *E*1	134	Combined	48 Hrs	29.3 ± 25.1	60.3 ± 24.2	22.8 ± 22.7	55.9 ± 23.7
08 − *E*2	30	Oil overlay	48 Hrs	21.3 ± 21.8	49.1 ± 26.5	15.0 ± 13.7	44.9 ± 24.1
08 − *E*2	90	No oil	48 Hrs	36.9 ± 24.1	66.3 ± 19.0	27.7 ± 22.8	58.5 ± 23.3
08 − *E*2	130	Combined	48 Hrs	32.0 ± 24.4	60.4 ± 23.1	24.4 ± 22.0	55.1 ± 23.6
09 − *E*1	32	Oil overlay	48 Hrs	9.7 ± 11.0	51.2 ± 24.0	8.6 ± 13.1	47.6 ± 26.1
09 − *E*1	91	No oil	48 Hrs	25.4 ± 22.4	62.2 ± 23.8	16.6 ± 17.8	58.3 ± 25.9
09 − *E*1	131	Combined	48 Hrs	22.6 ± 22.3	58.2 ± 24.5	14.8 ± 17.6	54.3 ± 26.4
09 − *E*2	30	Oil overlay	48 Hrs	49.1 + 14.0	6.8 + 2.0	43.5 + 11.0	5.0 + 1.0
09 − *E*2	95	No oil	48 Hrs	68.4 + 14.0	22.7 + 7.0	63.3 + 14.0	17.7 + 6.0
09 − *E*2	134	Combined	48 Hrs	61.9 + 10.0	20.6 + 4.0	56.1 + 10.0	14.9 + 4.0
